# The Management of Sick Children

**Published:** 1882-05

**Authors:** 


### THE MANAGEMENT OF SICK CHILDREN.

The vicissitudes necessarily incident to an out-door and primitive mode of life are never the first causes of any disease, though they may sometimes betray its presence. Bronchitis, nowadays perhaps the most frequent of all infantile diseases, makes no exception to this rule; a draught of cold air may reveal the latent progress of the disorder, but its cause is long confinement in a vitiated and overheated atmosphere, and its proper remedy ventilation and a mild, phlegm-loosening (saccharine) diet, warm, sweet milk, sweet oatmeal porridge or honey-water. Select an airy bed-room, and do not be afraid to open the windows. Among the children of the Indian tribes, who brave in open tents the terrible winters of the Hudson Bay Territory, bronchitis, croup and diphtheria are wholly unknown; and what we call "taking cold" might often be more correctly described as taking hot; glowing stoves, and even open fires, in a night nursery greatly aggravate the pernicious effects of an impure atmosphere. The first paroxysm of croup can be promptly relieved by very simple remedies: Fresh air and a rapid backward and forward movement of the arms, combined in urgent cases with the application of a flesh-brush or piece of flannel to the neck and upper part of the chest. Paregoric and poppy syrup stop the cough by lethargizing the irritability, and thus preventing the discharge of the phlegm, till its accumulation produces a second and far more dangerous paroxysm. These second attacks of croup—after the administration of palliatives—are generally the fatal ones. When the child is convalescing, let him beware of stimulating food and over heated rooms. Do not give aperient medicines; costiveness, as an after effect of pleuritic affections, will soon yield to fresh air and a vegetable diet.

